# Complex clinical and microbiological effects on Legionnaires’ disease outcone; A retrospective cohort study

**DOI:** 10.1186/s12879-016-1374-9

**Published:** 2016-02-10

**Authors:** Ariela Levcovich, Tsilia Lazarovitch, Jacob Moran-Gilad, Chava Peretz, Eugenia Yakunin, Lea Valinsky, Miriam Weinberger

**Affiliations:** 1 Infectious Diseases Unit, Assaf Harofeh Medical Center, Zerifin, 70300 Israel; 2 Microbiology Laboratory, Assaf Harofeh Medical Center, Zerifin, 70300 Israel; 3 National Program for Legionellosis Control and Public Health Services, Israeli Ministry of Health, 39 Yermiyahu St., 5th Floor, Jerusalem, Israel; 4 ESCMID Study Group for Legionella Infections (ESGLI), Basel, Switzerland; 5 Faculty of Health Sciences, Ben-Gurion University of the Negev. Ben-Gurion Boulevard, Beer-Sheva, Israel; 6 School of Public Health, Epidemiology, Sackler School of Medicine, Tel Aviv University, POB 39040, Tel Aviv, 69978 Israel; 7 Molecular Laboratory, Central Laboratories, Israel Ministry of Health, POB 34410, Jerusalem, 94467 Israel; 8 Sackler School of Medicine, Tel Aviv University, POB 39040, Tel Aviv, 69978 Israel

**Keywords:** *Legionella pneumophila*, Legionnaires’ disease, risk factors, mortality, diagnosis, logistic model, molecular typing

## Abstract

**Background:**

Legionnaires’ disease (LD) is associated with high mortality rates and poses a diagnostic and therapeutic challenge. Use of the rapid urinary antigen test (UAT) has been linked to improved outcome. We examined the association between the method of diagnosis (UAT or culture) and various clinical and microbiological characteristics and outcome of LD.

**Methods:**

Consecutive patients with pneumonia and confirmation of *Legionella* infection by a positive UAT and/or a positive culture admitted between the years 2006–2012 to a university hospital were retrospectively studied. Isolated *L. pneumophila* strains were subject to serogrouping, immunological subtyping and sequence-based typing. Variables associated with 30-day all-cause mortality were analyzed using logistic regression as well as cox regression.

**Results:**

Seventy-two patients were eligible for mortality analyses (LD study group), of whom 15.5 % have died. Diagnosis based on positive *L. pneumophila* UAT as compared to positive culture (OR = 0.18, 95 % CI 0.03–0.98, *p* = 0.05) and administration of appropriate antibiotic therapy within 2 hospitalization days as compared to delayed therapy (OR = 0.16, 95 % CI 0.03–0.90, *p* = 0.04) were independently associated with reduced mortality. When controlling for intensive care unit (ICU) admissions, the method of diagnosis became non-significant. Survival analyses showed a significantly increased death risk for patients admitted to ICU compared to others (HR 12.90, 95 % CI 2.78–59.86, *p* = 0.001) and reduced risk for patients receiving appropriate antibiotic therapy within the first two admissions days compared to delayed therapy (HR 0.13, 95 % CI 0.04–0.05, *p* = 0.001). *Legionella* cultures were positive in 35 patients (including 29 patients from the LD study group), of whom 65.7 % were intubated and 37.1 % have died. Sequence type (ST) ST1 accounted for 50.0 % of the typed cases and ST1, OLDA/Oxford was the leading phenon (53.8 %). Mortality rate among patients in the LD study group infected with ST1 was 18.2 % compared to 42.9 % for non-ST1 genotypes (OR = 0.30, 95 % CI 0.05-1.91, *p* = 0.23).

**Conclusions:**

The study confirms the importance of early administration of appropriate antibiotic therapy and at the same time highlights the complex associations of different diagnostic approaches with LD outcome. Infection with ST1 was not associated with increased mortality. Genotype effects on outcome mandate examination in larger cohorts.

## Background

*Legionella pneumophila*, especially serogroup 1 is an important cause of severe pneumonia which is usually acquired in the community and less commonly during hospitalization [1, 2]. *Legionella* pneumonia, also known as Legionnaires’ disease (LD), is characteristically a disease of middle age and elderly patients, and may be associated with a high mortality rate [3, 4]. Possible factors contributing to adverse outcomes include host susceptibility, appropriateness of therapy and possibly the virulence of infecting strain [5–7].

*Legionella spp.* are resistant to beta-lactam antibiotics, which constitute the most commonly prescribed agent for patients with pneumonia. The recommended treatment for infections due to *Legionella spp*. includes macrolides, quinolones and tetracyclines [1]. Hence, appropriate antibiotic therapy may be often delayed, especially if *Legionella* is not a priori considered the etiologic agent. Another obstacle for the timely administration of appropriate therapy is delayed diagnosis. Respiratory culture-based diagnosis, the gold standard for diagnosis, requires specialized media and expertise, has a long turnaround time (7-14 days), and is not routinely performed in many clinical laboratories [8]. Serologic studies mostly require paired samples obtained two weeks apart [8] and largely have been abondoned in recent years.

The introduction of the urinary antigen tests (UAT) for detection of *L. pneumophila* serogroup 1, the most common etiologic agent of LD, has revolutionized LD diagnosis. In the US, between the years 2000–2009 UAT was used to confirm LD diagnosis in 97 % of cases, culture in 5 % and serology or direct fluorescent antigen testing in <1 % [9]. Similarly in Europe the average detection rate by UAT was 79 % and 10 % by culture [10]. UAT allows rapid and timely diagnosis and has been linked to decreasing mortality trends in the US [2]. The sensitivity of the current tests for *L. pneumophila* serogroup 1 is in the range of 70-90 % with high specificity (95–100 %) [8]. Some tests are able to detect other serotypes with lower sensitivity [8]. While being rapid and readily available in many laboratories, the increasing dependency on UAT for diagnosis of LD may result in substantial under-detection rate reaching up to 40 % of cases [8, 11]. Lower rates of culture-based diagnoses may limit our ability to study the full scope of LD epidemiology, perform outbreak investigation and study the possible impact of *L. pneumophila* phenotype (particularly non-1 serogroups) or genotype and various clinical variables on LD. [2, 8, 12]. The rapidly evolving molecular diagnostic methods are increasingly used for LD diagnosis but still are less informative than culture-based methods [13].

LD is a challenge to public health authorities worldwide [14]. The incidence in the US is on the rise and has reached 1.15 per 100,000 population in 2009 [9]. In EU countries the incidence has been stable in recent years. The overall notification rate in 2012 was 1.1 per 100 000 inhabitants with an associated 10 % case fatality rate [15]. In Israel, *Legionella* infection is an important, although uncommon cause of community-acquired and nosocomial pneumonia [16–18]. In a recent national surveillance study the reported incidence was 0.5 to 0.9 cases per 100,000 population, corresponding to 38–63 annual case. The case fatality rate was 12.3 % [18]. The vast majority of cases (88 %) were documented by UAT, and only 7 % by culture [18].

In the present retrospective cohort study we aim to describe the epidemiology of LD in a single center in Israel where both *Legionella* cultures as well as UAT are available and routinely performed. We sought to examine the clinical and microbiological variables associated with outcome, with a focus on the method of diagnosis.

## Methods

### Patients and setting

Assaf Harofeh Medical Center is an 850-bed university affiliated hospital in Central Israel, serving a mainly urban population of circa 750,000. The Microbiology Laboratory serves as a national center for clinical diagnosis of *Legionella*. All consecutive patients with laboratory evidence of *Legionella* infection admitted between January 1, 2006 and December 31, 2012 were retrospectively identified from the Microbiology Laboratory records, and patients with diagnosis of pneumonia were included in the study. The diagnosis of pneumonia was based on appearance of a new infiltrate on chest radiogram and at least one of the following: fever ≥37.8^0^C or hypothermia ≤ 35^0^C, cough with or without sputum production, pleuritic chest pain, dyspnea, or abnormal findings on chest auscultation. LD was defined as a case of pneumonia with supporting laboratory evidence (either a positive *L. pneumophila* culture from any source or a positive UAT for *Legionella*). Children <18 years and patients with undetermined disease onset were excluded from the clinical analyses. All patients with positive cultures were eligible for microbiologic analyses.

We assumed that decisions on specific anti-*Legionella* treatment were made by the treating physician when a positive test result for *Legionella* was reported. The primary method of diagnosis was defined as either a positive UAT test or a positive *L. pneumophila* culture. When both were positive in the same patient—the primary method of diagnosis was determined based on the order of reported test results. The number of adult patients with the diagnosis of pneumonia during the study years was retrieved from the hospital archives using ICD-9-CM diagnoses codes 481–484 and 486 for denominator calculation.

### Data collection

Detailed data regarding patient demographics, underlying diseases, possible exposure, clinical presentation, clinical course, diagnostic workup, intensive care unit (ICU) admission, need for intubation and outcome were retrieved from medical charts and laboratory records. Underlying conditions and performance status was assessed according to the Charlson score [19].

Macrolides (roxithromycin 150–300 mg bid or azithromycin 500mg qd), quinolones (ciprofloxacin 400–500mg bid, levofloxacin 500–750 mg qd or moxifloxacin 400 mg qd) or tetracyclines (doxycycline 100mg bid) given orally or intravenously were considered appropriate antibiotic therapy for LD. Throughout the study years the recommended empirical institutional protocols for the treatment of community acquired pneumonia not requiring intensive ICU admission consisted of an intravenous third generation cephalosporin (ceftriaxone 1–2g qd) with a macrolide (roxithromycin or azithromycin) or a respiratory quinolone (levofloxacin or moxifloxacin, usually given orally), or a respiratory quinolone alone. For patients who were admitted to ICU the empirical treatment consisted of an intravenous third generation cephalosporin (ceftriaxone) with an intravenous macrolide (azithromycin) or a respiratory quinolone (levofloxacin or moxifloxacin).

### Laboratory methods

Respiratory specimens were routinely inoculated on blood agar, chocolate agar, Sabouraud dextrose agar and MacConkey agar media and commercial buffered charcoal yeast extract medium (BCYE) for *Legionella*. Blood cultures were processed by the BacT/Alert system (Organon Teknika, Durham, NC, USA). Growing isolates were identified as *Legionella* by Legionella Latex Agglutination Test (Oxoid, Basingstoke, UK), which allows differential identification of *L. pneumophila* serogroup 1 and serogroups 2–14 as well as the detection of seven other *Legionella* species which have been implicated in human disease. Specific identification of serogroups 2–14 was performed using monoclonal antibodies (m-TECH Inc., Milton, GA, USA). Immunological subgrouping was performed for representative strains using the Dresden Panel of monoclonal antibodies [20].

Urinary antigen testing was performed using a commercial ELISA-based kit (Bartels *Legionella* Urinary Antigen ELISA, Trinity Biotech, Bray, Ireland), according to the manufacturer’s instructions. Equivocal results were resolved by testing using a second commercial kit (BinaxNOW *Legionella* Urinary Antigen EIA, Alere, Orlando, FL, USA).

Molecular typing was carried out according to the Sequence-Based Typing (SBT) scheme for *Legionella pneumophila* (ESCMID Study Group for *Legionella* Infections, formerly EWGLI) [21, 22]. The allelic profiles and sequence types (ST) were determined using the *Legionella* Sequence Quality Tool [23] as well as the BioNumerics (Applied Maths, Sint-Martens-Latem, Belgium) software .

### Statistical methods

To study the association between all-cause 30-day mortality (a binary dependent variable) and various factors, univariate logistic models were initially applied. Significant (p ≤ 0.10) non-correlated factors were next included in multivariate logistic regression models to calculate odds ratio (OR) and 95 % confidence interval (CI).

Kaplan–Meier survival curves and univariate Cox regressions were used to compare time to death (all-cause mortality within 30 days from admission) between patients who received appropriate vs. inappropriate therapy within 2 days of hospital admission and those who were admitted or not to ICU. SPSS version 21 (SPSS Inc., Chicago, Il, USA) was used for all analyses.

### Ethical consideration

The study was approved by the local ethical committee at Assaf Harofeh Medical Center and patient’s anonymity was secured throughout the study

## Results and discussion

### Patients and outcome

**Fig. 1:**
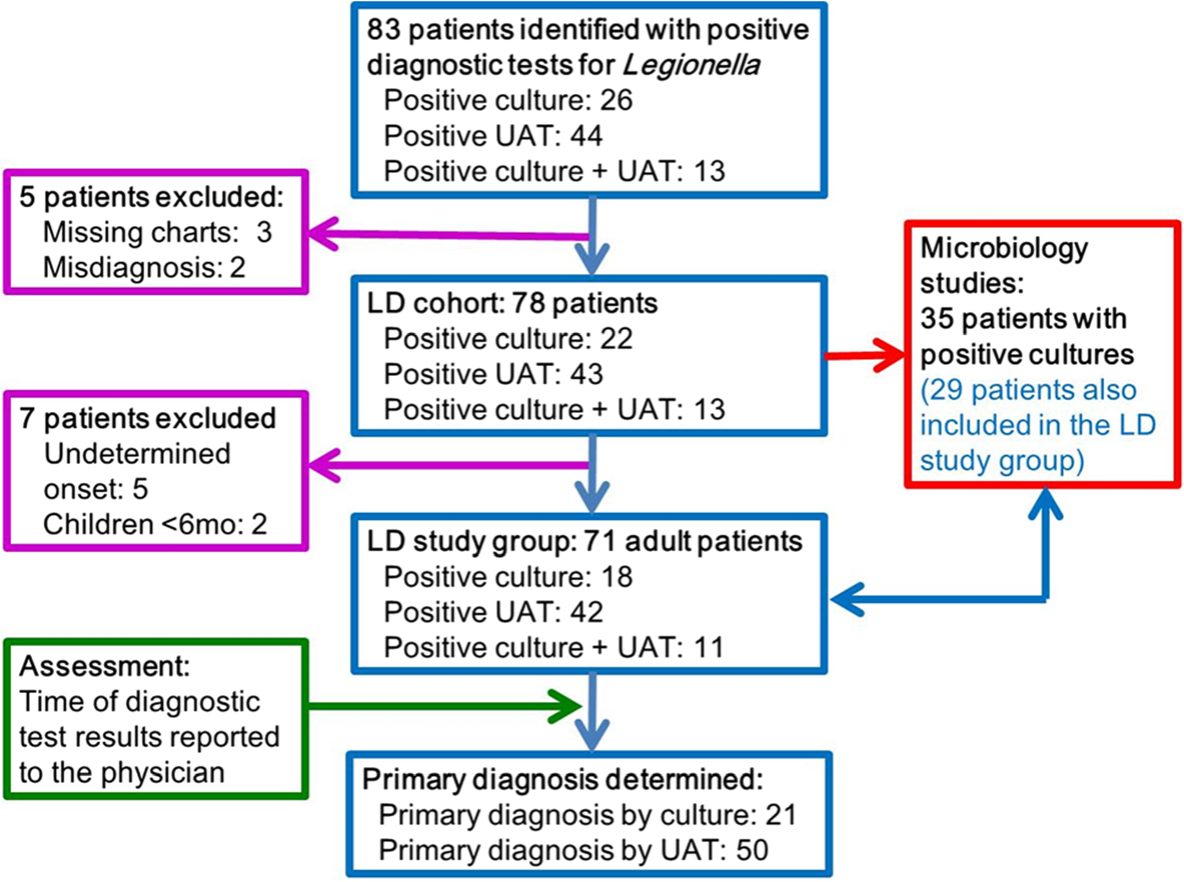
Flow chart depicting patient selection and inclusion in the analyzed groups. Abbreviations: *UAT* urinary antigen test, *LD *Legionnaires’ disease

During the study period, 83 patients were identified with positive *Legionella* culture (26 patients), positive UAT test (44 patients) or both (13 patients) (Fig. 1). Five patients were excluded due to missing charts (*n* = 3) or misdiagnosis (*n* = 2). Seventy eight patients fulfilled the selection criteria for LD and thus comprised the study cohort. Five adult patients with undetermined disease onset and two pediatric patients aged <6 months, who contracted humidifier-associated pediatric LD (reported elsewhere [24] ) were excluded from the mortality analyses. Six of these patients had positive cultures and were included in the microbiology analyses. Seventy one adult patients were thus eligible for LD mortality analysis, and will be referred to as the LD study group. During the study period 9,015 were discharged from the hospital with a diagnosis of pneumonia (average of 1,288 per year).

Diagnosis of LD in the study group was based on positive culture in 18 patients (25.4 %), positive UAT in 42 patients (59.2 %), and both in 11 patients (15.5 %). Altogether culture was positive in 29 patients (40.8 %). According to timing of laboratory reports it was judged that primary LD diagnosis was based on UAT results in 50 patients (70.4 %) and on culture results in the remaining 21 patients (29.6 %).

The characteristics of the patients included in the LD study group are presented in Table 1. The all-cause 30-day mortality rates for the LD study group were 15.5 % overall and 42.9 % in those admitted to ICU. In the univariate analyses (Table 1) high Charlson comorbidity index (OR = 1.33, 95 % CI 1.00–1.77, *p* = 0.05), diagnosis of leukemia (OR = 8.00, 95 % CI 1.63–39.35, *p* = 0.01), prior hospitalization within 30 days (OR = 5.14, 95 % CI 1.16–22.82, *p* = 0.03), and ICU admission (OR=18.00, 95 % CI 3.43-94.44, *p*=0.001) were positively associated with all-cause 30-day mortality. Primary diagnosis by UAT compared to diagnosis by culture (OR = 0.17, 95 % CI 0.004–0.68, *p* = 0.01) and administration of appropriate antibiotic therapy within two hospitalization days compared to delayed therapy (OR = 0.09, 95 % CI 0.002–0.37, *p* = 0.001) were negatively associated with mortality.

**Table 1:** Univariate logistic analyses, variables associated with all-cause 30-day in-hospital mortality

Parameters^a,^	Alive (*N* = 60)	Dead (*N* = 11)	OR, 95 % CI	P-value
Demography				
Age, years, mean ± SD	65.7 ± 15.5	74.6 ± 9.5	1.05, 1.0–1.10	0.08
Male sex^b^	42 (70.0)	8 (72.7)	0.88, 0.21–3.68	0.86
Predisposing conditions				
None^c^	7 (11.7)	0	-	1.00
Myocardial infarction^c,d^	21 (35.0)	5 (45.5)	1.55, 0.42–5.68	0.51
Congestive heart failure^c,d^	8 (13.3)	4 (36.4)	3.71, 0.88–15.62	0.07
Chronic pulmonary disease^c,d^	13 (21.7)	2 (18.2)	0.08, 0.15–4.19	0.80
Diabetes^c,d^	17 (28.3)	2 (18.2)	0.56, 0.11–2.87	0.49
Leukemia^c,d^	4 (6.7)	4 (36.4)	8.0, 1.63–39.35	0.01
Lymphoma^c,d^	3 (5)	2 (18.2)	4.22, 0.62–28.87	0.14
Solid tumor^c,d^	6 (10)	0	-	0.58
Metastatic cancer^c,d^	2 (3.3)	1 (9.1)	2.9, 0.24–35.07	0.40
Steroid treatment^c, e^	7 (11.7)	3 (27.3)	2.84, 0.61–13.30	0.19
Chemotherapy^c, e^	7 (11.7)	3 (27.3)	2.84, 0.61–13.30	0.19
Current smoker^c^	23 (38.3)	2 (18.2)	0.36, 0.07–1.80	0.21
Ever smoked^c^	35 (58.3)	6 (54.5)	0.86, 0.24–3.12	0.82
Hospitalization within 30 days^c^	6 (10)	4 (36.4)	5.14, 1.16–22.82	0.03
Charlson comorbidity index, mean ± SD	2.3 ± 2.1	3.7 ± 2.1	1.33, 1.00–1.77	0.05
Laboratory variables at admission				
Oxygen saturation, %, mean ±SD	92.1 ± 5.2	88.3 ± 10.7	0.92, 0.83–1.02	0.12
Neutrophil count < 1.0 x10^9^/L^c^	2 (18.2)^g^	7 (10.0)	2.4, 0.40–14.31	0.34
Lymphocyte count < 1.0 x10^9^/L^c^	38 (64.4)^g^	5 (45.5)	0.46, 0.13–1.69	0.24
Creatinine level, μmol/L, mean ± SD	123.8 ± 70.7	168.0 ± 106.1	1.60, 0.88–2.89	0.12
Bilateral infiltrates on chest X-ray^c^	14 (23.3)	4 (36.4)	1.88, 0.48–7.36	0.37
Primary diagnosis by positive UAT^f^	14 (23.3)	7 (63.6)	0.17, 0.04–0.68	0.01
Antibiotic therapy & outcome				
Appropriate therapy on day +1^c^	37 (61.7)	3 (27.3)	0.23, 0.06–0.98	0.05
Appropriate therapy on day +2 ^c^	52 (86.7)	4 (36.4)	0.09, 0.02–0.37	0.001
Delay in appropriate therapy administration, days, mean ± SD	0.9 ± 1.8	4.2 ± 5.6	1.34, 1.08–1.67	0.01
Quinolones started on day +1^c^	17 (28.3)	3 (27.3)	1.13, 0.26–4.80	0.87
Quinolones started on day +2^c^	23 (38.3)	4 (36.4)	0.92, 0.24–3.49	0.90
ICU admission^c^	12 (20.0)	9 (81.8)	18.00, 3.43–94.44	0.001
Invasive ventilation ^c^	10 (16.7)	8 (72.7)	13.33, 3.00–59.19	0.001

*Abbreviations*: *OR* odds ratio, *CI* confidence interval, *SD* standard deviation, *ICU* intensive care unit

^a^No.(%) unless otherwise stated

^b^Reference category: female sex

^c^Reference category: no

^d^Variables included in the Charlson comorbidity score

^e^Within 30 days preceding hospitalization

^f^Reference category: primary diagnosis by positive culture

^g^Missing data = 1

Four non-correlated variables and age were included in the first multivariate model (Table 2 multivariate model 1). Early administration of appropriate antibiotic therapy within two hospitalization days compared to delayed treatment (OR = 0.16, 95 % CI 0.03–0.90, *p* = 0.04) and primary diagnosis by UAT compared to cultures (OR = 0.18, 95 % CI 0.03–0.98, *p* = 0.05) were found as independent variables negatively associated with death at 30 days. ICU admission was considered an intermediate variable and was not included in the initial model.

**Table 2:** Logistic regression, multivariate models of variables associated with all-cause 30-day in-hospital mortality

Parameter	OR	95 % CI	P-value
Multivariate model 1			
Mean age, years	1.05	0.98-1.13	0.18
Hospitalization within 30 days^a^	1.48	0.19-11.37	0.71
Charlson comorbidity index	1.20	0.81-1.79	0.36
Primary diagnosis by positive UAT^b^	0.18	0.03-0.98	0.05
Appropriate therapy on day +2^a^	0.16	0.03-0.90	0.04
Multivariate model 2			
Mean age, years	1.06	0.95-1.18	0.31
Hospitalization within 30 days^a^	1.93	0.19-19.28	0.58
Charlson comorbidity index	1.37	0.88-2.12	0.16
Primary diagnosis by positive UAT^b^	0.88	0.12-6.81	0.91
Appropriate therapy on day +2^a^	0.04	0.004-0.55	0.009
ICU admission^a^	55.54	2.94-1049.35	0. 007

*Abbreviations*: *OR* odds ratio, *CI* confidence interval, *UAT* urinary antigen test, *ICU* intensive care unit

^a^Reference category: no

^b^Reference category: primary diagnosis by positive culture

ICU admission rates were found to be significantly higher in patients primarily diagnosed by culture (57.1 %) compared to UAT (18.0 %) (OR = 6.07, 95 % CI 1.97–18.73, *p* = 0.002). Notably cultures were submitted for all 21 patients admitted to ICU and for 32 of 50 patients (64.0 %) who were not. When ICU admission was added into the multivariate model as a covariate (Table 2 multivariate model 2) the primary method of diagnosis was no longer significant, while early administration of appropriate antibiotic within two hospitalization days remained significant.

**Fig. 2:**
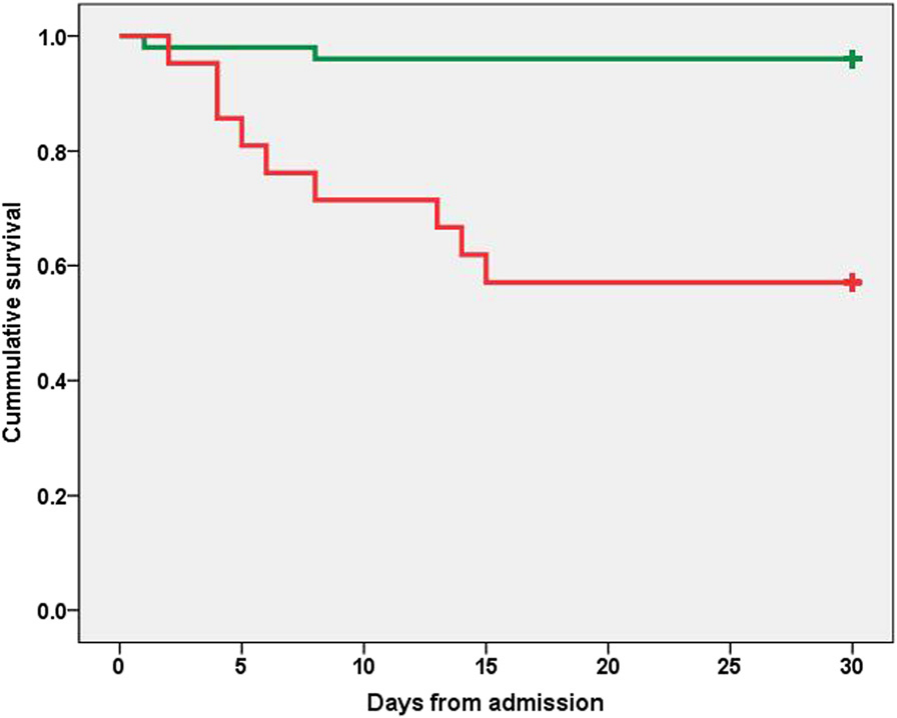
Survival of patients with Legionnaires’ disease as a function of intensive care unit admission. Legend to Fig. 2: Kaplan-Meier short survival curve (all-cause 30-day in-hospital mortality) for patients with Legionnaires' disease who were (red line) or were not (green line) admitted to the intensive care unit (HR = 12.9, 95 % CI 2.780–59.860, *p* = 0.001 by Cox regression). **+** Sign denotes censored. Abbreviations: *HR* Hazard ratio

**Fig. 3:**
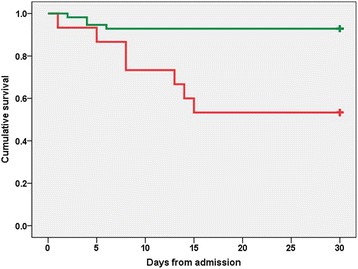
Survival of patients with Legionnaires' disease as a function of appropriate antibiotic therapy. Legend to Fig. 3: Kaplan-Meier short survival curve (all-cause 30-day in-hospital mortality) for patients with Legionnaires' disease who received (green line) or did not receive (red line) appropriate antibiotic therapy within the first two hospitalization days (HR = 0.134, 95 % CI 0.039–0.460, *p* = 0.001 by Cox regression). **+** Sign denotes censored. Abbreviations: *HR* Hazard ratio

Survival curves for all-cause 30-day mortality comparing patients by ICU admission or by appropriateness of therapy within the first two days of admission are presented in Figs. 2 and 3. The hazard ratios according to the Cox regression analysis were 12.90 (95 % CI 2.78–59.86, *p* = 0.001) and 0.13 (95 % CI 0.04–0.46, *p* = 0.001) for these two comparisons, respectively.

### Microbiologic results

Culture was positive for *L. pneumophila* in 35 of 78 (44.9 %) of the patients in the LD cohort as follows: *L. pneumophila* serogroup 1 in 28 (80.0 %) patients, serogroup 4 in 2 (5.7 %) patients, serogroup 3 and serogroup 8 in one (2.9 %) patient, each. The serogroup of 3 isolates could not be determined. Combined with the positive UAT results, 71 of 78 patients (91.0 %) were presumably diagnosed as having serogroup 1 infection (91.5 % of 71 patients in the LD study group). Importantly, the majority of positive cultures (23 of 35, 67.5 %) originated from patients who were intubated.

**Fig. 4:**
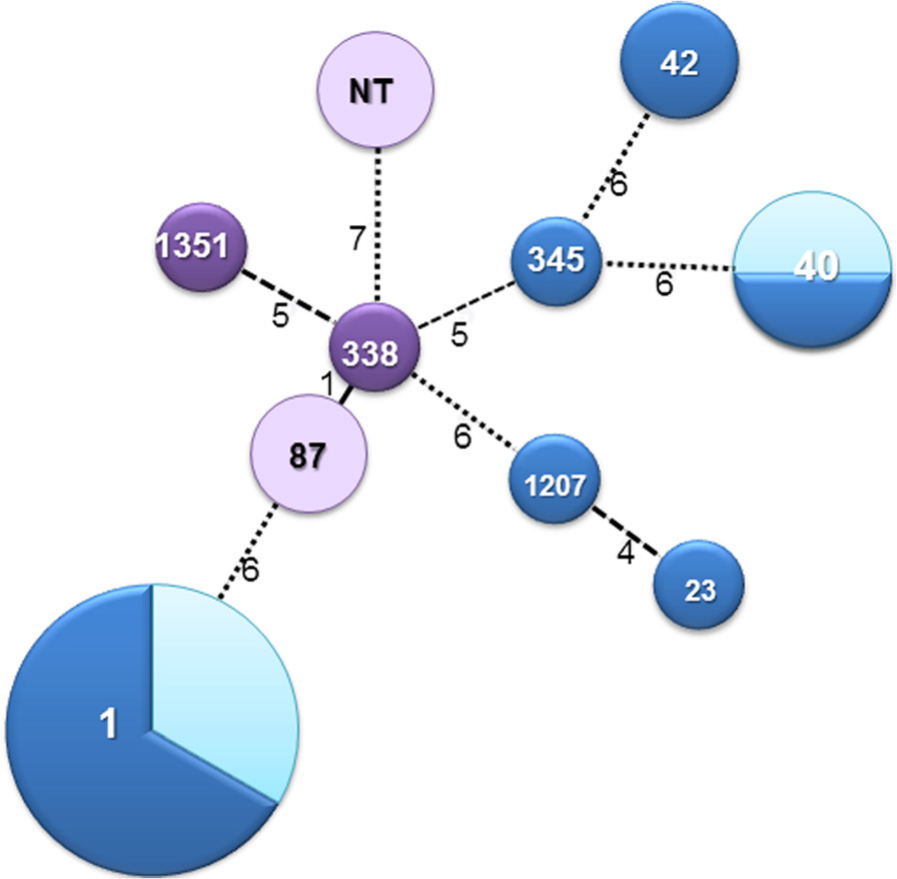
Genetic relatedness of the characterized *L. pneumophila* isolate*s* and associated mortality. Legend to Fig. 4: Minimum spanning tree showing the sequence types (ST) of *L. pneumophila* strains isolated from 30 patients with Legionnaires' disease. Blue circles represent serogroup 1 strains and purple circles represent the non-1 serogroups. ST number is shown inside the circle, and circle size is proportional to the number of strains assigned to each ST. Lighter shades of blue and purple denote fatal cases (all-cause 30-day in-hospital mortality). Line length connecting circles is proportional to the number of allele difference (in parentheses) between the defined STs. NT denotes nontypeable

Genotyping was performed on 30 of 35 (85.7 %) *L. pneumophila* isolates. Some of the isolates were included in a recent report from Israel [18]. The following STs were found: ST1 (15 cases, 50.0 %), ST40 (4, 13.3 %), ST42 (2, 6.7 %), ST87 (2, 6.7 %), and ST23, ST338, ST345, ST1207, ST1351 (one case, each). ST1207 and ST1351 were novel STs [18], and additional two isolates (6.7 %) were untypeable. The genetic relatedness of the characterized strains is shown in Fig. 4.

Immunological subtyping was performed for 13 isolates with known STs: all seven ST1 isolates belonged to OLDA/Oxford, and the two ST40 isolates belonged to the Allentown subgroup. The other four isolates were Allentown,ST23; Allentown,T1207, Benidorm,ST42, and Knoxville,ST345. Thus OLDA/Oxford,ST1 was the most common phenon responsible for 53.8 % of the characterized phenons and 23.3 % of all typed isolates.

All-cause 30-day in-hospital mortality rate for all 35 patients with positive cultures was 37.1 %. Five of 15 patients with designated ST1 died (33.3 %). Both patients with ST87 and both patients with nontypeable isolates died, as well two of the four patients with ST40 (Fig. 4). Among the patients included in the LD study group only 29 had positive cultures and 9 have died (31.0 %). ST was determined for 25 patients, and 11 were assigned ST1. Mortality rate among patients infected with ST1 genotype was 18.2 % compared to 42.9 % for those infected with non-ST1 genotypes (OR = 0.30, 95 % CI 0.05–1.91, *p* = 0.23). ICU admission rates among these patients were 36.4 % and 78.6 %, respectively (OR = 0.16, 95 % CI 0.03–0.92, *p* = 0.05).

## Discussion

We describe a cohort of patients with LD from a single center where *Legionella* UAT and cultures from respiratory specimens are routinely performed. This unique setup enabled us to assess the impact of diagnosis by UAT compared to culture on outcome. We found that diagnosis by UAT was associated with decreased mortality at 30 days when compared to diagnosis by a positive culture. However, when controlling for ICU admission, this association was no longer present, suggesting that the severity of the disease and invasive ventilation may have been confounding factors. Indeed, the majority of the patients in the LD study group primarily diagnosed by culture was admitted to ICU and was intubated implying that high quality sputum samples were more readily achievable in that patient group.

In a large epidemiologic study from the US, Benin et al. [3] described the impressive trend of decreasing mortality from LD concomitant with the increasing use of UAT for diagnosis. However, when looking at each subgroup separately, the mortality trend was significant only among persons diagnosed by culture, but not among those diagnosed by UAT. It was argued that UAT may have selected persons with less severe illness or hastened the initiation of appropriate antibiotics.

We found that administration of appropriate antibiotics within the first two hospitalization days was negatively associated with fatal outcome, even after controlling for ICU admission. This finding is in agreement with a recent large study of LD from Spain, showing that inappropriate empirical antibiotic therapy was an independent risk factor for severe outcomes (e.g. ICU admission or death) [25]. Early coverage of *Legionella* has proven beneficial in a recent large prospective study of combination therapy for moderate to severe community acquired pneumonia [26]. This can be achieved by adherence to the Infectious Diseases Society of America/American Thoracic Society consensus guidelines for the empirical therapy of community acquired pneumonia recommending the administration of combination therapy including a beta-lactam antibiotic plus an agent covering atypical organisms [27]. Despite the controversy regarding the added value of an agent covering ‘atypical’ pathogens in the combination regimens [28–33], its role in patients with LD has not been disputed [34]. Our institutional guidelines recommended combination therapy for empirical community acquired pneumonia therapy and specific anti-*Legionella* coverage for severe pneumonia. Unfortunately, the compliance with these guidelines was less then optimal. Only 56.3 % of the patients in the LD study group received appropriate antibiotic therapy on day 1.

All-cause 30-day in-hospital mortality rates in our LD study group reached 15.5 %. Comparison to mortality rates in other studies should be carried out with caution, taking into account differences in the patient mix, delay in appropriate antibiotic treatment and the methodology of outcome reporting. For example, in a recent large study including 215 hospitalized patients from a single center in Barcelona the in-hospital fatality rate was 6.1 % [25]. However, there were several important differences between the two studies: firstly, in the Spanish study the mean patients’ age was 58.2 years compared to 67.2 years in our study group. Secondly, patients with immunosuppressive conditions were excluded from the Spanish cohort. Thirdly, positive cultures were reported in only 22.7 % of the Spanish cohort compared to 40.8 % in our study group. Lastly, in-hospital fatality rate is not the same as all-cause 30-day mortality rate. The 2014 ECDC surveillance report [10] found a lower average case fatality rate of 9 % for cases acquired in the community and higher rates of 28 % for nosocomial cases. In addition, the majority of cases were diagnosed by UAT (79 %) and only 10 % by culture. On the other hand, in a recent national report from France [35] including 1,192 patients with culture-confirmed LD and a mean age of 59.4 years, the death rate of 18.6 % was closer to the rates reported in our study.

The proportion of serogroup 1 (80.0 %) among isolates obtained by culture in our study is similar to the global rates [10, 36]. The distribution of genotypes, with ST1 (50.1 %) and the OLDA/Oxford,ST1 phenon (23.3 %) the most frequently encountered isolates in our center, differed from many European localities. In a study from England and Wales [37] the leading genotypes from clinical isolates were ST47 (25.7 %) and ST37 (11.4 %), while ST1 accounted for only 4.8 % of isolates. However, ST1 was the leading genotype among environmental isolates (19.6 %). In another study from Belgium [38] the leading genotype among patient with community-acquired legionellosis was ST47, while ST1 was the most frequent isolate among nosocomial cases. In a recent French study the leading genotype was ST23 (19.8 %), while ST1 ranked third (9.1 %) [36]. Notably, in a study from Japan ST1 was the leading genotype (8.1 %) and OLDA,ST1 the most commonly encountered phenon [39].

Studies examining possible correlation between *Legionella* genotypes and clinical course or outcome of LD are scarce. In our study mortality rates among patients infected with ST1 was somewhat lower compared to the whole group of patients with positive cultures. Mortality rates were higher in patients with some other STs (100 % for STs 87 and nontypeable isolates and 50 % for ST 40), but the numbers in each group are too small for any generalization. A large epidemiologic study from France involving 1,090 patients and using pulsed field gel electrophoresis as the genotyping tool showed that mortality rates differed between genotypes, with Paris strain being most lethal [5]. Notably, the ‘Paris’ strain is in fact ST1 which was the most commonly encountered ST in our series. In a more recent study from the same group in France the following death rates were found: 30.9 % for ST1, 16.3 % for ST23, 13.2 % for ST47 and 18.6 % for all the other STs [35]. Although these studies suggest that ST1 is more virulent, however in our study ST1 was not associated with the highest mortality rates. One possible explanation is that less virulent strains may be associated with illness in immunocompromised patients and outcome in such patients may be grave due to delayed diagnosis or diminished immunity. Another possible explanation is that ST1 based on the SBT scheme may not be the same in all geographic localities. Using more discriminative next generation-based typing [40] in large epidemiologic studies may help to shed more light on this issue.

There are several limitations to our study. The study was of a retrospective nature, and may represent only a selected group of patients hospitalized with LD. For example, since UAT was not performed systematically in all admitted patients, it is possible that the treating physicians were more likely to look for LD in patients with more severe presentations. Such selection bias may result in higher ICU admissions and mortality rates. In addition, the study included a single center with a relatively small sample size of patients with positive cultures and genotype characterization.

## Conclusion

The present study confirms the importance of early administration of antibiotic therapy for improved outcome in hospitalized patients with LD. It also demonstrates the methodological challenges in studies attempting to assess the impact of the diagnostic strategy, such as rapid urinary test versus culture, on LD outcome. Diagnosis based on UAT may potentially shorten the time to the diagnosis and thus facilitate faster administration of appropriate therapy. However, the increasing use of empiric combination therapy for pneumonia covering atypical pathogens may diminish this impact. Diagnosis by culture is the gold standard and allows characterization of *L. pneumophila* isolates. Since culture is more readily achievable in patients who are intubated, reliance on culture may introduce a selection bias of sicker patients with worse outcome.

The study also provides preliminary data regarding the association of *L. pneumophila* molecular subtypes and clinical outcome. In contrast to some recent studies, ST1 did not stand out as the most virulent strain. These geographic variations may be further clarified by applying more refined molecular tools such as next generation sequencing- based typing schemes.

## Abbreviations

CI: confidence interval
HR: hazard ratio
ICU: intensive care unit
LD: Legionnaires’ disease
OR: odds ratio
SBT: Sequence-Based Typing
UAT: urinary antigen test
